# International assessment results on non-NIOSH approved respirators by the national personal protective technology laboratory (NPPTL): a data note

**DOI:** 10.1186/s13104-024-06800-0

**Published:** 2024-06-06

**Authors:** Kwai Yu Tsui, Yu Wang, Simon Ching Lam

**Affiliations:** 1 School of Nursing, Tung Wah College, 31 Wylie Road, Homantin, Kowloon, Hong Kong SAR China; 2 Nursing Department, Affiliated Hospital of Zunyi Medical University, Guizhou Province, China; 3 Present Address: School of Nursing, Zunyi Medical University, Guizhou Province, China

**Keywords:** Filter resistance, Filtration efficiency, Leakage, The national personal protective technology laboratory (NPPTL), National institute for occupational safety & health (NIOSH), N95 respirator, COVID-19, Respirator shortage, TSI Model 8130A, TEB-APR-STP-0059

## Abstract

**Objectives:**

Due to the COVID-19 pandemic and the shortage of the National Institute for Occupational Safety & Health (NIOSH)-approved N95 respirators, the Food and Drug Administration granted an Emergency Use Authorization to allow the use of non-NIOSH approved respirators provided that these respirators must undergo tests by a protocol of TEB-APR-STP-0059, similar methods of NIOSH standard testing procedure. This initiative safeguards the quality of respirators and the effectiveness of occupational protection. The dataset of all the testing results could benefit further analysis of COVID-19 infection rates in relation to different types of N95 respirators used and identify potential correlations of various test parameters in the testing system for validation. The analysis enhances understanding of the quality, effectiveness, and performance of N95 respirators in the prevention of respiratory infectious transmission and develops improved occupational safety measures.

**Data description:**

The dataset was transformed, transcribed, and compiled from the official testing data of non-NIOSH-approved N95 respirators reported in the NIOSH website under the Centers for the Disease Control and Prevention in the United States. The dataset included details of 7,413 testing results of N95 respirators (manufacturer, model, and maximum and minimum filtration efficiency) and test parameters (flow rate, initial filter resistance, and initial percent leakage). Supplementary items were added to increase the availability of data analysis and enhance the interpretability of the assessments of the quality of N95 respirators.

## Objective

An N95 respirator (also named filtering facepiece respirators, FFRs) is a type of disposable, negative-pressure, and air-filtering facepiece mask with or without filtering valve. In contrast to cartridge-based elastomeric half-mask respirators or powered air-purifying respirator (PAPR), the respirator under study is a kind of particulate-filtering tight-fitting facepiece masks that are specifically designed to provide a highly secure fit to the face and effectively filter out airborne particles [1, 2]. This respirator also meets the N95 classification set by the U.S. National Institute for Occupational Safety and Health (NIOSH) and can filter out at least 95% of airborne particles with a mass median aerodynamic diameter of 0.3 μm [2]. The World Health Organization and the Center for Disease Control and Prevention (CDC) mandatorily recommend healthcare professionals to wear N95 respirators in clinical settings to reduce the spread of infectious respiratory diseases during the aerosol-generating procedures [1, 3].

At the beginning of the ongoing COVID-19 pandemic, the supply of NIOSH-approved N95 respirator was insufficient worldwide. In this regard, the Food and Drug Administration has permitted the use of non-NIOSH-approved respirators with an Emergency Use Authorization (EUA). Non-NIOSH-approved N95 respirators must undergo testing (using the Protocol of STP-0059 similar to the NIOSH standard testing procedure) to ensure sufficient occupational protection [3].

Prior to the COVID-19 pandemic, the literature focused on several key aspects related to N95 respirator usage, including the user-seal-check, fit rate, and real-time leakage. Improper face-seal fit and failed user-seal-check of the N95 respirator led to leakage and thus failed to provide respiratory protection against particles or infectious respiratory diseases [4–6]. However, the quality of N95 respirators, specifically its filtration efficiency, is rarely reported. Filtration efficiency is the verification of the particles being captured by the fibrous filtration media of N95 respirators when in use; that is, an N95 respirator may fail to provide protection with a low filtration efficiency due to material quality despite its guaranteed proper face-seal fit. NIOSH has presented the test results of non-NIOSH-approved N95 respirators in a format of images that was unable to conduct any investigation and analysis. These valuable data deserve a transcription to a format that facilitates further statistical analysis.

The sampled respirators were tested by the National Personal Protective Technology Laboratory (NPPTL) under CDC in the United States. The NPPTL estimated only the particulate filtration efficiency of the respirator by using a modified test plan of TEB-APR-STP-0059, and the respirator samples were non-NIOSH approved ones. The NPPTL offers an assessment of the filtration efficiency of respirators certified by a foreign regulatory authority, apart from NIOSH. During the COVID-19 pandemic, such assessment was used to evaluate the filtration efficiency of non-NIOSH approved respirators. The study provides gathered and organized raw data obtained by the NPPTL on filtration efficiency, initial filter resistance, and leakage of N95 to increase the availability of data and enhance the interpretability of the assessment regarding the quality of protection imparted by non-NIOSH-approved N95 respirators. It helped to ensure that healthcare workers were provided with adequate respiratory protection despite the respirator shortage at that time [7].

## Data description

The dataset was established from the official assessment of non-NIOSH-approved N95 respirators conducted by NPPLT between 2020 and 2021, and the test results (in the format of an image) were posted on the NIOSH website. The features and testing protocol of TEB-APR-STP-0059 are summarized as below [8]:

The necessary equipment and materials include filter tester TSI Model 8130–8130 A, a microbalance, type A or E glass filters, a timer, 2% sodium chloride solution, a temperature and humidity chamber, a respirator holder, and a data acquisition system or a thermal printer. The equipment must be calibrated and checked as per the calibration procedure established. The respirator and respirator’s filter cartridge are tested for particle penetration. If the filter cartridge are not separated from the body of the respirator, then precautions are taken to seal the exhalation valves to prevent leakage, which could potentially affect the results of filter penetration measurements. During the testing, the respirators are challenged by sodium chloride aerosol at 25 ± 5 °C with relative humidity of 30%±10%. The aerosol should not exceed 200 mg/m^3^. The challenge flow rate is adjusted according to the respirator configuration. The tested respirator is mounted and sealed on the holder to prevent leakage by chemical glue or mechanical mold. A sample of 20 respirators from each batch are tested. The instructions in the protocol ensure that individuals conduct the test process accurately and determine whether the effectiveness of respirators achieves the established requirement or not.

## Data construction

The TEB-APR-STP-0059 protocol used in this dataset measured several parameters (can be interpreted as internal/system control) to assess respirator filtration efficiency performance. These parameters include flow rate (liters per minute), initial filter resistance (mmH_2_O), initial and maximum percent leakage (%), and filtration efficiency (%). Flow rate is the airflow rate or the amount of air drawn through the filter of the respirator being tested and should be kept constant or within a minimum variation. Initial filter resistance indicates the initial pressure difference across the respirator filter through the rated air volume and is correlated with filtration efficiency within the same batch of samples [9–11]. Initial and maximum percent leakage indicates the percentage of air that passes through the micropores between filter’s fiber instead of going through the filter. A higher percentage of leakage indicates a less effective respirator. Filtration efficiency (%) measures the effectiveness of the respirator or its ability to capture particles (including virus or bacteria). This percentage indicates the proportion of particles filtered by the respirator. All these parameters are important to determine the creditability of the testing system and hence the quality of the tested respirators [11].

Apart from the above-mentioned raw data, supplementary items were added to the dataset including (S1) “FDA emergency use authorization (EUA),” (S2) “more than 15% difference between maximum and minimum filtration efficiency,” (S3) “maximum or minimum filtration efficiency lower than 80%,” (S4) “maximum filtration efficiency (%),” and (S5) “minimum filtration efficiency (%)” to enrich the content of data and enhance the interpretability of the assessments and the quality of protection of N95 respirators [12]. The dataset provides a useful record and resource for further retrospective analysis of COVID-19 infection rates among various N95 respirators used. Furthermore, the correlations of different parameters posed an implication for testing industries as a reference. A total of 7,413 testing results were transformed and transcribed from images into Excel data (Table 1). Only 1,430 (19.3%) N95 respirators were approved with an EUA from FDA. Of the 7,403 available tests (excluded 10 tests due to missing data), the initial filterer resistance was significantly and moderately correlated with the maximum filtration efficiency (*r = 0.435, p < 0.001*). A total of 1,963 testing results were then selected and extracted for further analysis because of (S2) fluctuated filtration efficiency and (S3) poor filtration efficiency. For these selected testing results, the initial filtering resistance was significantly and high-moderately correlated with the maximum filtration efficiency (*r* = 0.625, *p* < 0.001), consistent with reports in the literature [10, 11].

**Table 1:** Overview of dataset

Label	Name of data file/dataset	File types (file extension)	Data repository and identifier (DOI or accession number)
Dataset 1	NIOSH nonapprovedrespirator data	Excel (.xlxs)	Mendeley Data (10.17632/k8236t72h7.1) [12]

### Limitations

- Non-NIOSH-approved respirators tested using the modified test plan of the NIOSH standard test procedure (STP-0059) cannot be considered as equivalent to N95 respirators tested with STP-0059.
- NIOSH cannot control the supply and distribution of respirators certified by a foreign regulatory authority.
- The testing results did not provide all the system parameters, including the number of 0.3 μm particles upstream and downstream, face velocity of particles, and pressure of mixer.
- Calibration of the measuring device in this dataset was not shown, so a traceable method of calibration and its standards cannot be confirmed.

## Data Availability

The data described in this Data note can be freely and openly accessed on Mendeley Data under 10.17632/k8236t72h7.1. Details and links to the data are presented in Table 1 and Reference [12].

## Abbreviations

NIOSH: National Institute for Occupational Safety and Health
CDC: Centre for Disease Control and Prevention of the United States
NPPLT: The National Personal Protective Technology Laboratory
LPM: Liters Per Minute
